# Spermatic cord tunnel Penrose drainage for cephalad-tracking Fournier’s gangrene: a case report

**DOI:** 10.1093/jscr/rjag934

**Published:** 2026-10-19

**Authors:** Sepideh Garrosi, Necial Marcelin, Ingrid Paredes, Marlon Pastrana

**Affiliations:** Florida International University, Herbert Wertheim College of Medicine, 11200 SW 8th Street, Miami, FL 33199, United States; American University of Antigua, College of Medicine, University Park, Jabberwock Beach Road, PO Box W1451, Coolidge, Antigua and Barbuda; Department of General Surgery, Larkin Community Hospital, 7031 SW 62nd Avenue, South Miami, FL 33143, United States; Baptist Health Homestead Hospital, 975 Baptist Way, Homestead, FL 33033, United States; Department of General Surgery, Larkin Community Hospital, 7031 SW 62nd Avenue, South Miami, FL 33143, United States

**Keywords:** Fournier gangrene, surgical technique, spermatic cord drainage, wound reconstruction, dermal regeneration template

## Abstract

Fournier's gangrene tracking cephalad along spermatic cords complicates source control and soft tissue reconstruction. We describe a 61-year-old male with poorly controlled diabetes who presented with extensive scrotal necrotizing fasciitis tracking superiorly to the suprapubic region. Following wide excisional debridement, bilateral Penrose drains were passed through the spermatic cord tunnels and brought out through dependent perineal counter incisions. This technique provided effective through-and-through drainage of the cephalad tracts while limiting abdominal tissue loss. Deep wound cultures uniquely grew methicillin-resistant *Staphylococcus aureus* as a monomicrobial organism. Following serial debridements, a dermal regeneration template was applied to the scrotal and perineal defect, followed by staged split-thickness skin grafting. Complete coverage and structural preservation were achieved without the need for urinary or fecal diversion.

## Introduction

Fournier's gangrene is a rapidly progressive necrotizing soft tissue infection of the perineum and genitalia that spreads along fascial planes and carries substantial mortality [1–3]. While the disease typically remains localized to the scrotum and perineum, infection can uncommonly extend cephalad along the dartos, Scarpa's, and Colles's fascia and along the spermatic cords toward the lower abdominal wall [4, 5]. Superior tracking complicates initial source control because the tract must be effectively drained without converting it into an extensive, highly morbid open suprapubic wound bed.

Following control of the necrotizing process, the resulting extensive soft tissue loss requires structural reconstruction. Dermal regeneration templates generate a highly vascularized neodermis that supports delayed skin grafting and serves as an effective flap-sparing option, though experience remains limited in complex genital defects [6–8]. We present a unique case of Fournier's gangrene tracking along bilateral spermatic cords, successfully managed with through-and-through cord tunnel Penrose drainage and staged reconstruction using a dermal regeneration template.

## Case report

A 61-year-old male with a history of poorly controlled insulin-dependent diabetes mellitus, hypertension, and hyperlipidemia presented with a 1-day history of scrotal swelling (left greater than right), left-sided foul-smelling drainage, and skin discoloration. On arrival, he was afebrile (37.5°C) and hemodynamically stable (blood pressure 102/69 mmHg, heart rate 86 bpm) but demonstrated severe hyperglycemia (614 mg/dL), leukocytosis (white blood cell count 18.3 × 10^9^/L), and lactic acidosis with a lactic acid level of 3.0 mmol/L. Initial liver biochemistry was notable for an elevated alkaline phosphatase of 216 unit/L and an elevated total bilirubin of 1.4 mg/dL. Physical examination revealed a significantly distended, edematous, and tender scrotum with necrotic skin changes, foul-smelling purulent drainage, and palpable crepitus tracking superiorly into the bilateral groins and suprapubic region (Fig. 1). Preoperative axial computed tomography demonstrated extensive subcutaneous emphysema involving the scrotum and perineum with cephalad extension into the bilateral inguinal regions and lower suprapubic abdominal wall (Fig. 2). A clinical diagnosis of necrotizing soft tissue infection was made, empiric broad-spectrum intravenous antibiotics (cefepime, clindamycin, and vancomycin) were initiated, and the patient was taken emergently to the operating room.

**Figure 1:**
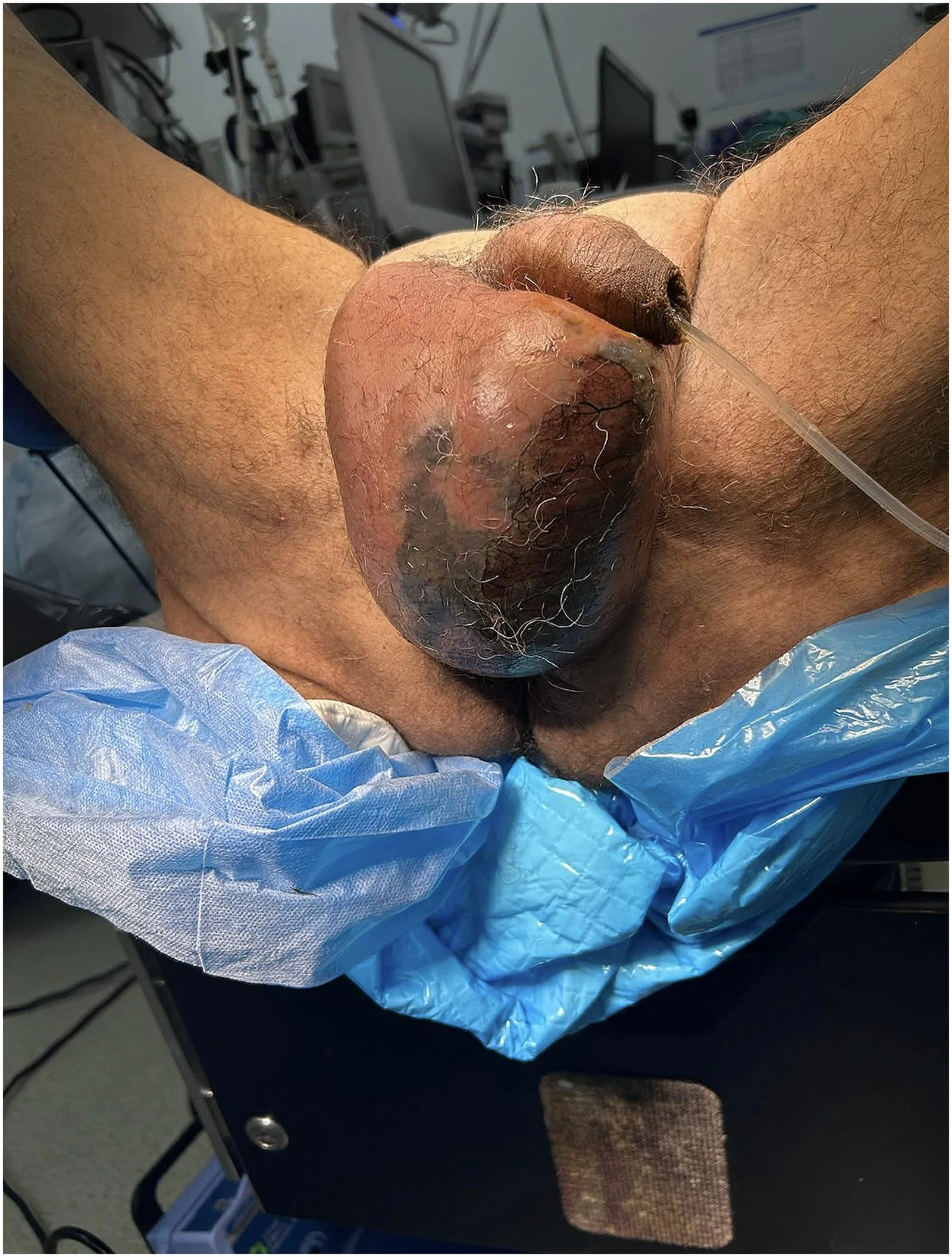
Initial preoperative clinical presentation demonstrating marked scrotal edema, skin discoloration, necrotic changes, and extensive swelling consistent with Fournier’s gangrene.

**Figure 2:**
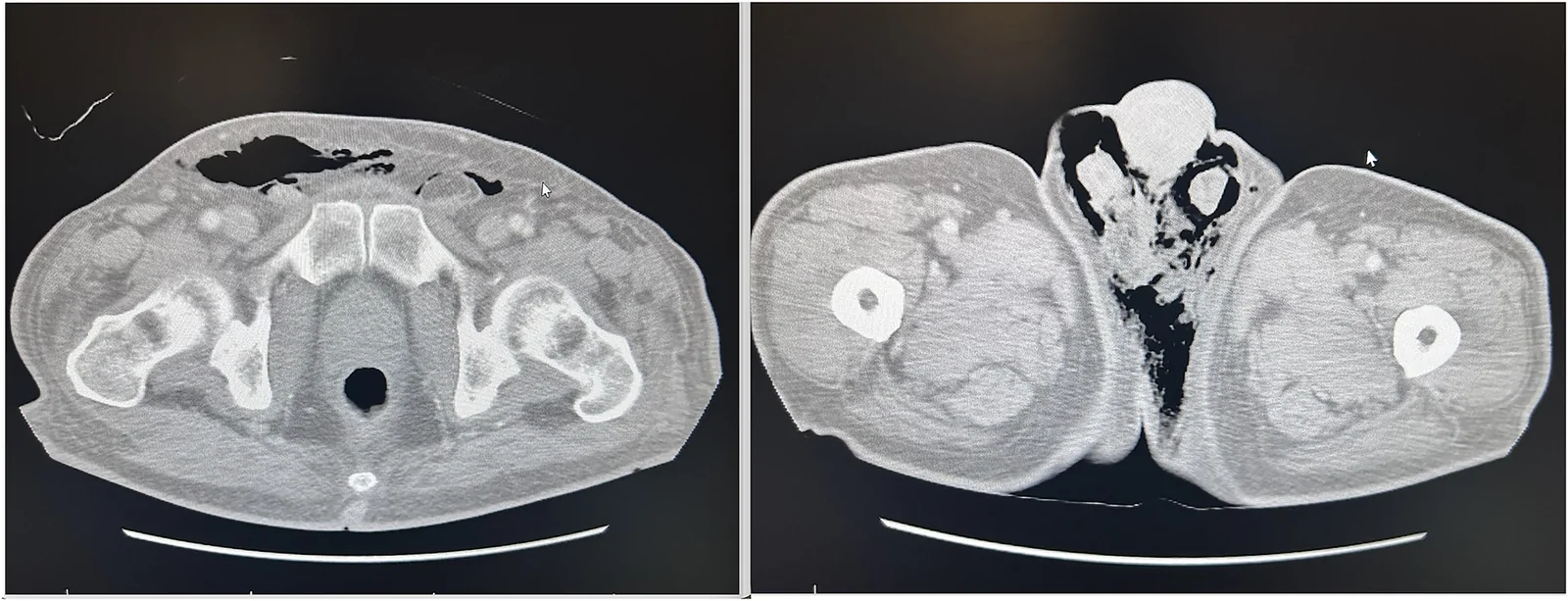
Preoperative axial computed tomography demonstrating the anatomical extent of Fournier's gangrene. (a) Subcutaneous emphysema involving the lower suprapubic and anterior abdominal wall soft tissues, demonstrating cephalad extension of the infection. (b) Extensive subcutaneous emphysema involving the scrotum and perineum.

At the index operation on April 11, wide excisional debridement of necrotic scrotal, perineal, dartos, and fascial layers produced a 12.1 × 12.2 cm defect with an estimated blood loss of 10 mL. Extensive air and grayish purulent fluid were found tracking superiorly along both spermatic cords into the suprapubic region. The testes were found to be viable and were preserved. To establish source control of the cephalad tract without extensive suprapubic tissue loss, small suprapubic incisions were made, and Penrose drains were passed bilaterally through the inguinal canal and spermatic cord tunnels, brought out through dependent counter incisions in the perineal wound bed to facilitate continuous through-and-through drainage (Fig. 3). Wound microbiology from the procedure grew methicillin-resistant *Staphylococcus aureus*, and antibiotic therapy was targeted accordingly.

**Figure 3:**
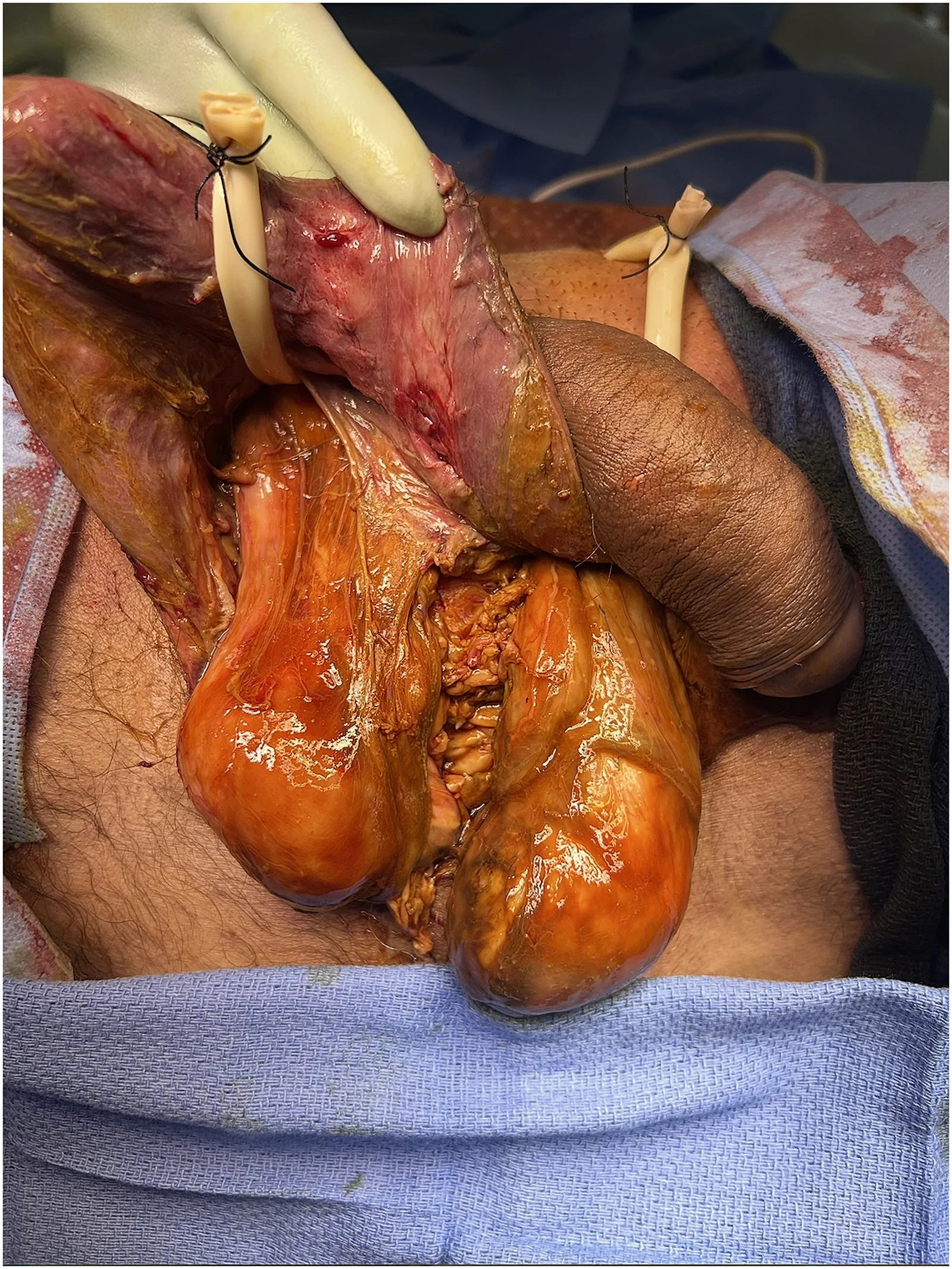
Intraoperative appearance following wide excisional debridement, demonstrating preservation of both testes and bilateral Penrose drains passed through the spermatic cord tunnels for continuous through-and-through dependent drainage.

The patient was admitted to the intensive care unit and returned to the operating room on April 14 and April 17 for serial surgical debridement. On April 14, sharp excisional debridement of a 12.1 × 2.2 cm area was performed, which included a 5 × 5 cm area of midline scrotal necrosis and a 3 × 3 cm area of additional necrotic subcutaneous fat and fascia in the perineal body; a significant purulent fluid collection lateral to the left spermatic cord was also aspirated and drained with a total estimated blood loss of 5 cc. On April 17, a 12.1 × 2.2 cm excisional debridement down to and including fascia was performed to resect a 3 × 3 cm area of new necrotic tissue on the right scrotal skin edge and associated subcutaneous necrosis; necrotic tissue involving the left epididymis was also encountered and sharply removed with an estimated blood loss of 5.0 mL. Surgical pathology of the excised necrotic scrotal tissue confirmed the definitive diagnosis of Fournier's gangrene.

The rectum and urethra were identified and carefully protected via a Foley catheter; no suprapubic catheterization or diverting colostomy was required. By April 21, the infection was eradicated and a healthy, bleeding granulation bed was established. Following a final 4.1 × 12.2 cm debridement of local skin necrosis and fibrinous tissue over the surgical bed (estimated blood loss 5 cc), a 10.1 × 12.5 cm Integra dermal regeneration template was operatively secured circumferentially to the scrotal and perineal skin edges using interrupted 2–0 chromic sutures. The bilateral suprapubic Penrose drains were successfully removed during this same procedure (Fig. 4). The patient tolerated the procedure well and was eventually discharged home on hospital day 12. He subsequently underwent successful staged application of a split-thickness skin graft harvested from the left thigh (Fig. 5). At the 6-week follow-up, the graft was completely incorporated without infection or structural complications, preserving native voiding, continence, and bilateral testicular viability (Fig. 6).

**Figure 4:**
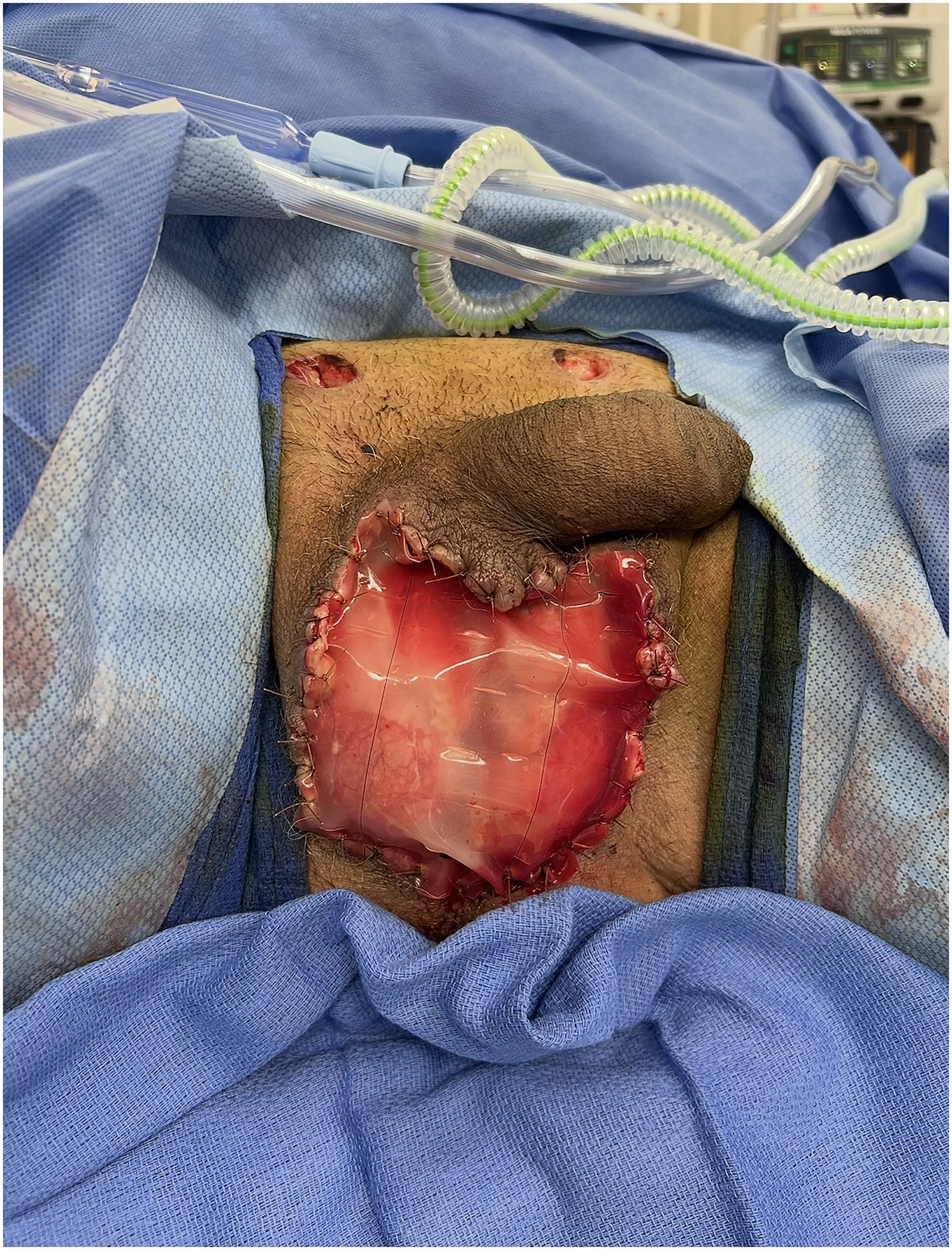
Dermal regeneration template application. The bilayer silicone-glycosaminoglycan matrix (Integra) is operatively secured and contoured seamlessly across the granulating scrotal defect.

**Figure 5:**
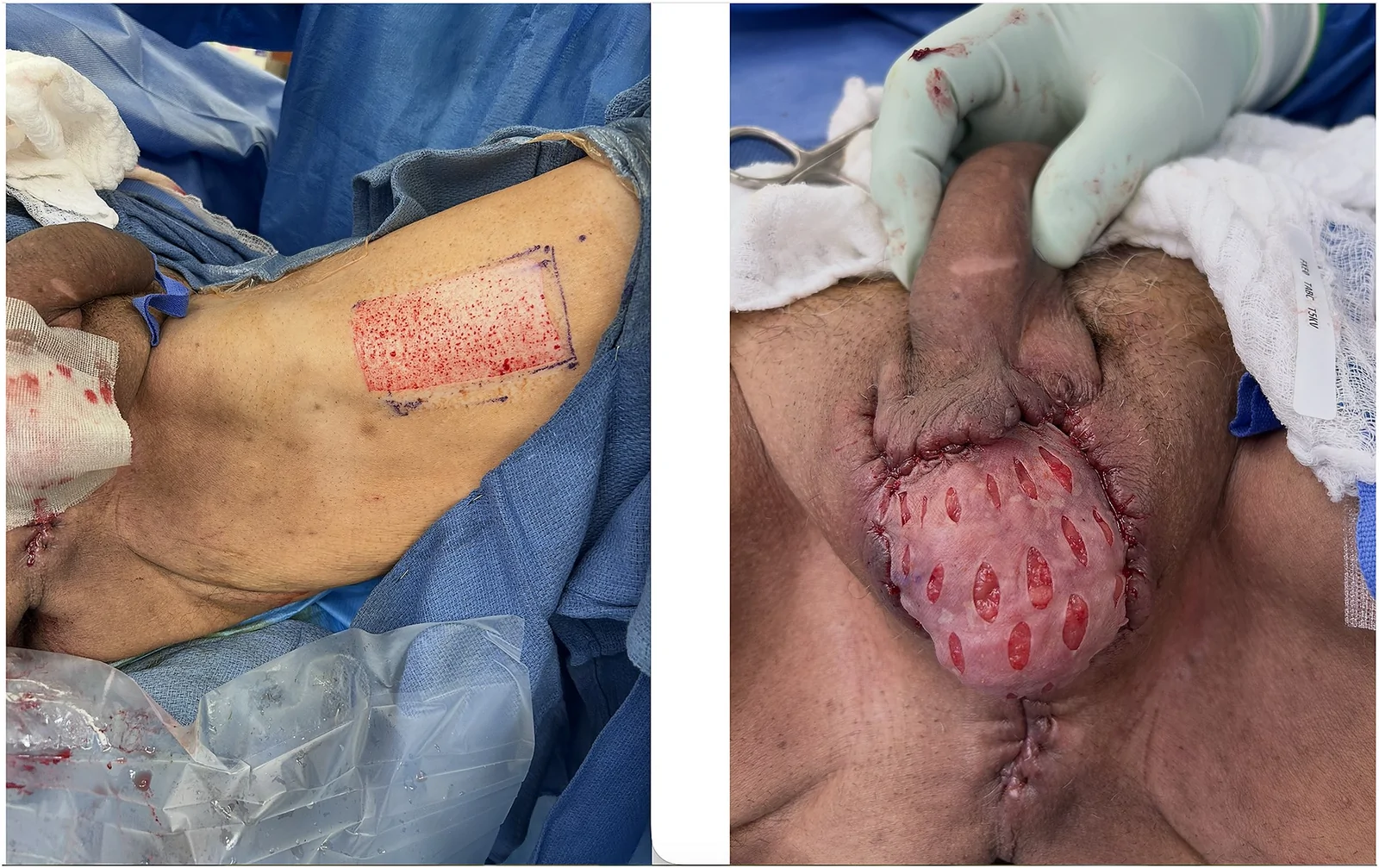
Staged split-thickness skin graft harvested from the left inner thigh and applied to the reconstructed scrotal and perineal wound bed.

**Figure 6:**
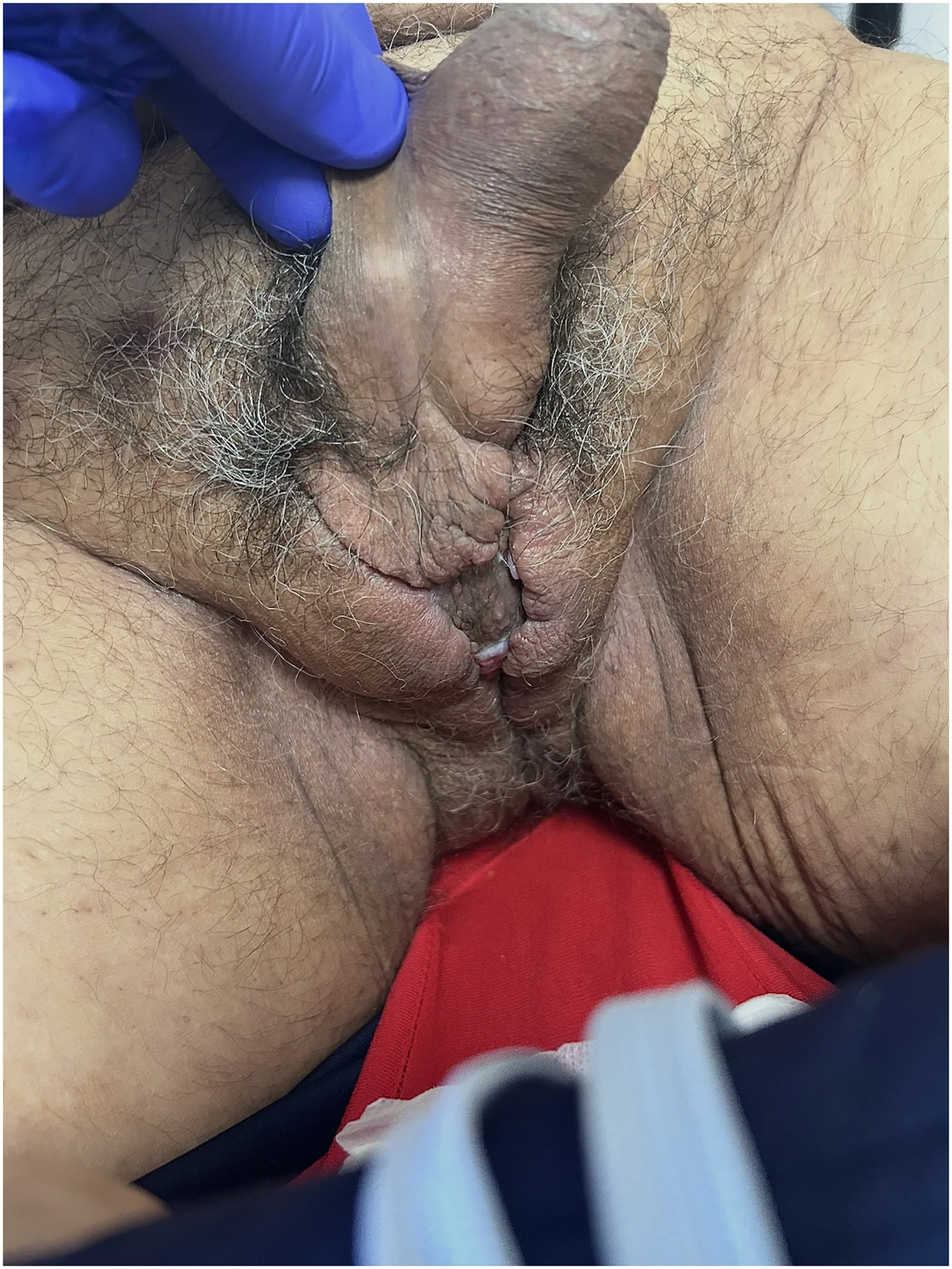
Clinical evaluation at 6-weeks follow-up demonstrating complete, stable epithelialization of the split-thickness skin graft with full anatomical preservation of the testes.

## Discussion

Fournier's gangrene remains an acute surgical emergency where survival depends heavily on the speed and completeness of operative debridements [1–3]. The distinguishing feature of this case was the bilateral cephalad extension along the spermatic cords to the suprapubic region. This tracking forces a difficult choice between creating a large, open suprapubic abdominal wound, or risking inadequate source control. Utilizing loop or seton drainage via Penrose drains passed directly through the compromised cord tunnels out to dependent perineal counter incisions provided continuous through-and-through clearance, successfully controlling the superior infection tract while actively preventing lower abdominal tissue loss and avoiding highly morbid abdominal incisions [4, 5].

Functional preservation remains a core tenet of modern perineal reconstruction. Routine urinary or fecal diversion via suprapubic catheters or diverting colostomies does not improve survival outcomes and introduces separate structural morbidities [8, 9]. By avoiding routine diversions and utilizing the classic reconstructive ladder, the surgical team optimized recovery [9, 10].

Dermal regeneration templates provide an extracellular matrix scaffold over vulnerable structures that cannot support immediate skin grafts, conforming seamlessly to the contour of the testes [10, 11]. Deploying the template in a staged fashion after achieving a clean granulation bed facilitated rapid graft incorporation and provided an excellent, flap-sparing reconstructive alternative for extensive external genital defects [11, 12].
